# Candidate and non-candidate genes in behavior genetics

**DOI:** 10.1016/j.conb.2012.07.005

**Published:** 2013-02

**Authors:** Jonathan Flint, Marcus R Munafò

**Affiliations:** 1 Wellcome Trust Centre for Human Genetics, University of Oxford, Roosevelt Drive, Oxford, United Kingdom; 2 School of Experimental Psychology, University of Bristol, 12a Priory Road, Bristol BS8 1TU, United Kingdom

## Abstract

In this review we discuss recent developments in psychiatric genetics: on the one hand, studies using whole genome approaches (genome-wide association studies (GWAS) and exome sequencing) are coming close to finding genes and molecular variants that contribute to disease susceptibility; on the other candidate genes, such as the serotonin transporter, continue to dominate in genetic studies of brain imaging phenotypes and in protracted searches for gene by environment interactions. These two areas intersect, in that new information about genetic effects from whole genome approaches, should (but does not always) inform the single locus analyses.

## Highlights

► Common variants of small effect contribute to psychiatric disease. ► Rare *de novo* mutations occur in the exons of patients with schizophrenia and autism. ► Effect sizes of loci influencing brain size are comparable to those of other phenotypes. ► The literature of imaging genetic studies contains many false positives. ► Studies on gene by environment interaction are mostly underpowered.

**Current Opinion in Neurobiology** 2013, **23**:57–61

This review comes from a themed issue on **Neurogenetics**

Edited by **Ralph Greenspan** and **Christine Petit**

For a complete overview see the Issue and the Editorial

Available online 8th August 2012

0959-4388/$ – see front matter, © 2012 Elsevier Ltd. All rights reserved.

**http://dx.doi.org/10.1016/j.conb.2012.07.005**

## GWAS and exome sequencing

Whole genome approaches address the vexed question of the genetic architecture of psychiatric disease: that is to say, how many loci are involved, how common are the variants, and what is the distribution of their effect sizes? A very practical concern drives this seemingly arcane question, namely, will it be possible to find genes in which mutations are the main, if not the sole, predisposing genetic cause? Finding even one such gene would provide a starting point for investigating disease pathogenesis because it is now relatively straightforward to model highly penetrant alleles, whereas it is not clear how to model a small effect locus that acts as part of a polygenic system to exert its effect on a phenotype.

Using linear mixed models (an increasingly popular methodology), Visscher and colleagues recently estimated the proportion of variance in liability to schizophrenia attributable to single nucleotide polymorphisms (SNPs) genotyped on commonly used arrays [1^••^]. These SNPs are chosen for typing because they are relatively common (allele frequencies greater than 5%). The researchers were able to put a lower limit on the genetic contribution: about one-quarter of the variance in liability was explained by common variants, refuting claims that common variants have only a small role in the genetic cause of schizophrenia. Conversely this means the remaining variation in susceptibility could be attributable to rare variants.

Studies that have sequenced the exomes of patients with schizophrenia and autism provide a bottom up answer to the same question: rather than assessing the impact of common variants, they search for rare coding variation. Two studies of schizophrenia, one of 14 trios (two unaffected parents and one affected child) [2^•^], the other of 53 sporadic cases, 22 controls and their parents, both reported *de novo* mutations in patients [3^•^]. The smaller study reported 4 non-sense mutations (out of a total of 15), significantly higher than the expected rate, while the larger study identified a ratio of 32 non-synonymous mis-sense mutations to 2 synonymous mutations, again significantly elevated over expectations.

Four papers recently reported exonic mutations in autism [4^•^,5^•^,6^•^,7]. Rates of non-sense mutations were elevated, though only modestly so. More autism patients were sequenced than in the schizophrenia studies, so an important question was whether mutations ever occurred in the same gene. This is a different question from that of asking whether coding mutations are more common in patients than controls. And it is an important question, because finding recurrent mutations could lead to the identification of a gene, or genes, causally implicated in the disease. Taken together, in all three studies, 18 genes with two functional mutations were found. However, by chance about 12 genes would be expected to have more than one mutation, and the enrichment was not quite statistically significant (*P* = 0.063) [5^•^].

Taking this observation further, Daly and colleagues pointed out that the results are consistent with a polygenic model in which spontaneous coding mutations increase risk between 5 and 20 fold [5^•^]. This finding is important because it demonstrates how the genetic architecture of psychiatric disease consists of a continuum. As Visscher argues, the dichotomy of rare variant of large effect versus common variant of small effect is specious, since the frequency and effect size of the alleles that increase susceptibility range across a continuum [8^•^].

One important conclusion that emerges from both GWAS and sequencing studies is that there are no common variants of large effect. To be precise, common variants that increase the chance of disease 1.3 fold or more are extremely unlikely to exist [9]. Similarly, for quantitative phenotypes, the expected effect sizes are less than 0.5% of phenotypic variance (for example, between 0.02 and 0.2% for each variant that contributes to variation in height [10]).

Four papers reinforce the generality of this conclusion [11^•^,12^•^,13^•^,14^•^]. All four deal with a field known as imaging genetics, that is the study of association between genetic variants and phenotypes obtained from structural and functional imaging of the brain (almost all studies are of human brains and the majority employ magnetic resonance imaging modalities). An important conclusion to emerge from these papers is that the genetic loci influencing imaging genetic phenotypes “have comparable effect sizes to those observed in other genome-wide association studies of complex traits” [11^•^]. To take one example, the rs10784502 marker is associated with 0.58% of intracranial volume per risk allele [11^•^]. The implications of this finding, and the other insights into the genetic architecture of behavior we have discussed, become clear when we turn to look at the second focus of our review.

## Candidate gene studies: brain imaging and G × E

In this section we discuss developments in two areas, first brain imaging and then gene by environment interactions (G × E for short). For many years psychiatric geneticists have had difficulties establishing robust associations between disease phenotype and allelic variant, leading some to argue that it would be better to work with phenotypes where the genetic architecture consists of loci of larger effect. Proponents of this uncontroversial proposition have suggested that neuroimaging phenotypes have the requisite property: that genetic effects on brain structural and functional variation are necessarily larger. The claim is based on the assumption that some phenotypes (often called endophenotypes) are biologically closer to the site of genetic variation (measures of mRNA would be an extreme example) and therefore the impact of genetic variation must be larger. Thus one study of just twenty-eight subjects reported an association between variation in amygdala activation and variation in a length polymorphism of the serotonin transporter gene (5-HTTLPR) [15]. The ‘short’ allele at this frequently typed polymorphism has a frequency of about 30% in European populations, and is thus a typical common variant, whose effect size on complex phenotypes we would expect to be small (explaining less than one percent of the variation in a quantitative measure). The short allele is reported to lower transcriptional efficacy (hence reduce levels of serotonin transporter protein). To obtain the degree of significance reported in the 2002 paper, the locus must explain about 28% of phenotypic variance (95% confidence intervals 15–53%). This effect size is indeed much higher than anything reported from genetic analyses of disease phenotypes, but is it likely to be true? Given the results of the imaging GWAS [11^•^,12^•^,13^•^,14^•^], the answer is that it is almost certainly not.

This finding needs emphasizing since it contradicts the results of a meta-analysis of the effect of a functional Val158Met (rs4680) polymorphism in catechol-O-methyltransferase (*COMT*) on neural endophenotypes [16]. That paper tested the hypothesis that “neural intermediate phenotypes are indeed more penetrant than behavioral ones” and reported a significant association between the *COMT* variant and test of ‘prefrontal activation’. The size of the effect was Cohen's *d* = 0.73. For those not familiar with this measure of effect size, it is the approximately the same as 12% of the variance, much larger than that found in the GWAS mentioned above.

Why the discrepancy? Is it really possible that many of the studies are false positives? Now this hypothesis can be tested, by determining whether the rate of positive findings is consistent with the reported effect sizes [17]: in other words, we can ask, given what the literature tells us about the effect size, how many positive reports should we expect to find? Ioannidis applied this test to structural brain imaging findings and observed 142 statistically significant findings among 461 studies, while the average power of these studies indicated that we should expect only 78.5 significant findings. This difference was itself significantly different [18^•^]. A recent meta-analysis investigating the effect of the serotonin transporter on amygdala activation provides the necessary data to test for the excess of false positives in imaging genetics studies [19]. Applying this, we find 11 statistically significant findings when 5.5 are expected (*P* = 0.027).

We turn next to discuss developments in the field of gene by environment interaction, focusing on publications involving the serotonin transporter. For those unfamiliar with this story, a brief reminder that the most highly cited paper in neuroscience in 2003 was the observation from a longitudinal study that possession of the ‘short’ allele of the 5-HTTLPR only increased the risk of developing depression in the presence of adverse life events [20]. This is an example of a gene by environment interaction (G × E), which opened the door to detecting many more such effects in studies that measured both environmental and genetic predisposition. While quantitative genetic studies indicated strongly that G × E existed in aggregate [21], this was the first demonstration that it could be detected at a single locus.

The hope was that studies of G × E, using carefully phenotyped individuals, might yield robust results that could be replicated. Unfortunately, the field has not developed in this way. In the last few years three meta-analyses of the literature have been published, and they reach opposite conclusions: two found no evidence for an interaction [22,23] while one concluded that there was an effect [24]. The view taken by the authors of the positive G × E meta-analysis is that the effect of G × E is broad: “rather than focus on a specific class of studies, we sought to perform a meta-analysis on the entire body of work assessing the relationship between 5-HTTLPR, stress, and depression”. In other words they incorporate more environmental effects and outcomes than envisaged even by the authors of the original study.

Additional findings over G × E at the serotonin transporter continue to accumulate, developing in two directions. One is the incorporation of additional sequence variants at the locus itself. Following the discovery that a single nucleotide polymorphism within one of the long alleles of the repeat means there is an ‘Lg’ allele with lower transcriptional efficacy (functionally therefore behaving like the ‘short’ allele) researchers now report G × E with additional alleles. However, justification for testing these additional alleles is weak. There has been no systematic investigation of the variants that contribute to expression variation in the transporter gene, but testing the effect of 55 SNPs distributed in a 100 kb window surrounding the serotonin transporter locus, as well as the length polymorphism made two important observations [25]. First, two SNPs in linkage disequilibrium explained 50% of variation in transcript abundance; the 5-HTTLPR contributed only 20%. Second, the Lg allele did not significantly contribute to variation. Thus we still lack comprehensive analysis of the relationship between functional variants at the 5-HTTLPR locus and phenotypic variation.

The second development is the increasing diversity of phenotypes that are being tested: these include quality of maternal parenting [26], affective state during marriage [27], risky sexual behavior [28], childhood emotionality [29], job satisfaction [30], perceived racial discrimination [31], adult unresolved attachment [32], and gaze bias [33]. A common feature of all these studies is the relatively small sample size: all except one [30] use samples of less than a thousand, and sometimes less than one hundred subjects [32]. Given the now well established main effect sizes discussed above, it seems unlikely, even allowing for the large effects observed in an interaction analysis, that any of these studies is sufficiently well powered to detect an effect.

The debate over G × E at the serotonin transporter locus is now considerably polarized [34^•^,35^•^,36^•^] but two recent papers are worth highlighting. First, Duncan and Keller used the pattern of publications to infer an excess of positive findings in the G × E literature [37^•^]. Their argument is that publication bias can be detected as a higher rate of positive results among novel findings compared to replication attempts, since journals preferentially publish positive findings for a novel genetic association. Second, one study replicated the design of the 2003 paper: a longitudinal study of a birth cohort of 1265 children born in New Zealand and studied from birth to the age of 30 [38]. The authors point out that “both studies have been conducted in the same geographic region (the South Island of New Zealand) over a similar historical period (1970–2010); both have gathered repeated-measures data on multiple sources of stress and adversity over the life course including: stressful life events, child abuse and trauma, exposure to inter-parental conflict, unemployment, violence victimisation and similar measures; and both have gathered measures of mental disorders using DSM criteria from adolescence into adulthood.” After testing 13 stress measures and 4 outcomes for G × E effects between number of 5-HTTLPR short alleles, the authors find “5 of the 52 results were statistically significant” (none become so if a correction for multiple testing is taken into consideration) but noted that “all significant tests of gene × environment interactions suggested that increasing numbers of ‘s’ alleles led to reduced sensitivity to stressful events” (i.e. the opposite direction to that predicted from the original study).

It is unlikely that either finding will dampen the enthusiasm for studies of G × E involving the serotonin transporter. Panagiotou and Ioannidis recently reported that the authors of primary studies are more likely to believe that a strong association exists than methodologists (referring here to the authors of meta-analyses) [39]. Evidence alone seems not be enough to change people's minds. In a careful presentation of the statistical difficulties inherent in the detection of small effects, Gelman and Weakliem [40] discuss what transpires when a theory can explain findings in any direction. They quote Jeremy Freese who describes this sort of argument as “more ‘vampirical’ than ‘empirical’—unable to be killed by mere evidence.”

## References and recommended reading

Papers of particular interest, published within the period of review, have been highlighted as:

• of special interest
•• of outstanding interest
